# Reconsidering Governance Models to Strengthen Rural Healthcare

**DOI:** 10.1007/s11673-025-10484-x

**Published:** 2025-10-23

**Authors:** F. McDonald, C. Malatzky, D. Sedgwick Fincher, L. Elliott, K. Purser

**Affiliations:** 1 Faculty of Law, Victoria University of Wellington, P.O. Box 600, Wellington, 6140 New Zealand; 2 Australian Centre for Health Law Research, Queensland University of Technology, Brisbane, Australia; 3 QUT Centre for Justice, Centre for Decent Work and Industry, School of Public Health and Social Work, Queensland University of Technology, GPO Box 2434, Brisbane, Queensland 4000 Australia; 4 School of Law, Queensland University of Technology, GPO Box 2434, Brisbane, Queensland 4000 Australia; 5 Australian Centre for Health Law Research, School of Law, Queensland University of Technology, GPO Box 2434, Brisbane, Queensland 4000 Australia

**Keywords:** Health governance, Rural health governance, Participatory governance, Rural healthcare, Health justice

## Abstract

This paper argues for a re-balancing of decision-making power in health systems away from metrocentric and technocratic norms and a fundamental re-shaping towards democratic and participatory models of governance. These changes are understood as critical to the objective of improving healthcare access and delivery in regional, rural, and remote communities. In articulating this argument, we highlight the highly political nature of how we structure and govern healthcare delivery, which is imbued with value judgments. We critique currently dominant neo-liberal approaches to healthcare governance that frame the “voices” of local communities as optional inputs into the system, rather than active agents in decision-making processes. From health justice and spatial justice perspectives, public participation in governance may be an important factor to enable improved access to regional, rural, and remote health services and to promote community self-determination.

## Introduction

Globally there are concerns that health services provided to people who live in regional, rural, and remote places are often inadequate in terms of access, timeliness, and sometimes in terms of quality, in comparison to services available to those in major cities or highly urbanized areas (Australian Institute of Health and Welfare 2022; Legislative Council New South Wales Portfolio Committee No. 2-Health 2022; Scheil-Adlung, 2015; World Health Organization, 2021). Initiatives to address these inequities often focus on the recruitment and retention of health professionals in rural places, including training and financial incentives. Less attention is paid to the implications of governance models on access to care in regional and rural communities (McDonald and Malatzky 2023; Simpson and McDonald 2017).

One dominant factor that has been identified by inquiries into rural healthcare (Legislative Council New South Wales Portfolio Committee No. 2-Health, 2022; Rural Health Services Review Committee 2015) by rural residents, and which is also reflected in the literature, is the perception amongst those who live in regional and rural places of what Pesut et al. (2011, 6) describe as “decision-making by [urban] strangers, at a distance.” This practice may result in tensions between community expectations or wishes and what health services are available to that community, when, and how, with real consequences for the residents of those communities. This is a question about where decision-making sits geographically, and who makes the decisions (technocrats, politicians, and/or local communities), and what informs their decision-making.

The last quarter of a century has also seen the centralization of healthcare systems to urban or metropolitan areas where regional or area hubs may be utilized to service “other” places. Centralized service provision was perceived as being more cost-effective (Bourke et al. 2012) and it also addressed concerns about healthcare quality (i.e. that healthcare quality would be threatened or compromised in non-urban and/or non-specialist settings) (Atmore et al. 2023). However, as some scholars have attested, these arguments are underpinned by a narrow definition of healthcare quality. Importantly, centralization has impacted the ability of persons residing in regional, rural, and remote places to access services locally, requiring them to travel greater distances, if they have access to transport and/or the ability to use it, with corresponding economic and social costs (Maclaren et al. 2024). These decisions may also occur without the necessary recognition of the economic and social benefits for communities associated with local provision and/or the negative impacts of such services being withdrawn (Atmore et al. 2023; Simpson and McDonald 2017). Some communities feel a sense of ownership in the local health service in their region but may struggle to find anything other than tokenistic ways to express that (Atmore et al. 2023; Simpson and McDonald 2017).

Considering the strengths and limitations of governance models is therefore a necessary part of thinking about reforms to make health services more equitable across different place contexts. To do this, we need to assess political, economic, and cultural power differentials that have influenced health governance (Palmquist 2020; Shawar and Ruger 2020). Structuring health systems is an inherently political process driven by questions of both who and what we value, as well as who we vest with decision-making power, what we empower them to do, who they involve and what they use in making decisions (Palmquist 2020; Simpson and McDonald 2017). Thus, how we structure and govern regional, rural, and remote health services is an inherently political question. Our research explores the gap in the literature on how the governance of health services might be a factor that could positively impact on access to healthcare in regional, rural, and remote places. This is consistent with Malatzky et al.’s (2020a, 2020b, 1) argument for “place-sensitive research” in rural settings to enable “… increasing understanding and acceptance of why place matters in broader societal and political domains” (Malatzky et al. 2020a, 2020b, 4). It is also consistent with Simpson and McDonald’s call for bioethical analysis into governance and policy structures that shape service delivery, access, and care in rural and remote areas (Simpson and McDonald 2017). Place-based research is also important in developing policy for rural communities (Atterton and Glass 2021).

This paper argues that decision-making power needs to both be rebalanced away from metro-centric norms (McDonald and Malatzky 2023) and become increasingly democratic to enable rural residents greater decision-making power around health service design and delivery to ensure care provision is responsive to their needs and priorities. Palmquist (2020, 394) argues that “all groups of people with common needs should be able to shape the health and economic systems that affect them.” In our current context, this requires critique of dominant approaches to governance where communities are treated as optional “voices” that can input into the system through pre-determined (and controllable) mechanisms, rather than key agents who can act with influence in the system. Clarification on the reasons why public participation is important for enabling improved access to regional, rural, and remote healthcare in theoretical terms is thus a crucial preliminary step.

## Democratic and Participatory Governance

Integral to the concept of health justice is “collective oversight through inclusive, democratic governance … to guide stewardship of health care resources and ensure equitable distribution of the burdens and benefits of public investments in health” (Wiley et al. 2022, 637). However, many nations across the world have been influenced by neo-liberal approaches to governance. Health system governance has long been a focus of the global health community but from a government centred perspective (Meessen 2020). This framing also often emphasizes decision-making driven by economic and/or other technical concerns, a view that can undermine citizenship and democracy (Barr 2008; Horton and Lynch-Wood 2020; Simonet 2019). Under this framing, citizens are no longer seen as active participants in co-governance with rights and responsibilities but rather become consumers who should, or may, be consulted with by designated external, and often technocratic, decision-makers but have no real role in exercising the levers of power (Palmquist 2020). Thus, these approaches often emphasize “voice” (the option to provide input, usually through pre-existing mechanisms) rather than “power” (the actual ability to make decisions, influence outcomes, and embed forms of accountability) (Palmquist 2020).

This technocratic emphasis aligns with that of an audit society wherein organizations, including health services, come to be constituted by their auditability; a key technique through which the logic of neo-liberal governance can be advanced (Power 2021), and organizational capabilities to engage consumers in strategy and governance depleted (Farmer et al. 2018). It also reflects the arguments of Peeters (2013) and Farmer et al. (2015) that these forms of “community consultation” constitute what Foucault conceptualized as the government (the ways in which our conduct is directed), in this case, of rural communities, “to structure the possible field of action” (Foucault 1982, 790). Consequently, decision-making is centralized in designated political or technocratic actors, as well as geographically; in the metropole where urban norms are privileged and the power of rural places relegated (Malatzky and Couch 2023; McDonald and Malatzky 2023). Hence, some argue that public participation enhances democracy by redressing power imbalances between citizens and the public vis-à-vis politicians, bureaucrats, and professionals (Arnstein 1969). Mullen et al. (2011) note that while technical and economic issues are relevant, they are not the complete answer. Decisions about healthcare governance are also decisions about values, which are inherently place contingent (Simpson and McDonald 2017). For example, how the community-accessibility of maternity services in rural communities is balanced with efficiency and perceptions of the service’s ability to comply with quality and safety standards is an inherently political activity with value judgements at its core.

There are a variety of formulations of participatory governance. Within these, an important conceptual distinction exists between utilitarian and empowerment models of participation (Morgan 2001). A utilitarian model regards participation as a means to an end; the end being to achieve a project’s aims effectively and efficiently by persuading people to engage with and contribute time, resources, and labour to an externally developed programme or plan. An empowerment model, in contrast, regards participation as an end in and of itself. In other words, the goal is to empower people as individuals and communities to become integral to the decision-making process. As Blacksher (2013, 7) puts it “the empowerment model treats participation as a social process through which the community takes responsibility for diagnosing problems, identifying opportunities and strategies for change, and taking collective action to improve health and social wellbeing.” An empowerment model, by definition, provides power to local actors: something that can be constraining or enabling (Bourke et al. 2012).

A move to an empowerment model also serves a democratic end—to maximize citizen engagement in governance, which also supports the enactment of values such as solidarity as well as meaningful, active, and free participation (Australian Human Rights Commission, n/d) and advances human rights, especially the right to the “highest attainable standard of physical and mental health” (ICESCR, art 12, 1966), and the sustainable development goal of good health and well-being (UN Department of Economic and Social Affairs 2015). Further, citizen engagement addresses the aspect of realizing justice that relates to accountability. The capacity for both prospective and retrospective accountability is enhanced by broadening the role of public participation in local health service governance (Sharpe 2004). As Sharpe (2004, 14) notes, “prospective responsibility is oriented to the deliberative and practical processes involved in setting and meeting goals.” So why is this concept of participatory governance particularly important to the delivery of health services in regional, rural, and remote places?

## Governance of Regional, Rural, and Remote Health Services

Inequities in access to health services and health outcomes are experienced globally by regional, rural, and remote residents (Glynn et al. 2023; Scheil-Adlung 2015; World Health Organization 2021). In the health context, the enterprise of a universal public health system builds on the value of solidarity. Citizens within a nation-state pool and re-distribute taxpayer funds to support each other to access the resources needed for health, including access to health services, and achieve broadly congruent health outcomes (Prainsack and Buyx 2015). This sense of solidarity and trust in the system can be undermined if a segment of the population does not benefit in an equitable way compared to others (Simpson and McDonald 2017). There are of course many factors that account for disparities in health outcomes, including inter alia genetic and occupational risk profiles. But one crucial factor is how systems are governed and whether that structure works to meet the needs of communities across different place contexts. In this sense, there has been growing recognition that some population health outcomes can be influenced by the ways in which health services are funded, designed, and delivered (Tenbensel and Silwal 2023).

In its broadest sense, justice includes participatory democracy, the belief that citizens and communities should be given opportunities to participate, more broadly than through consultation, in the governance of systems and structures that are important to them (Simpson and McDonald 2017). Participation is framed as having intrinsic value as an ethical practice consistent with enabling broad understandings of democracy (Palmquist, 2020; Simpson and McDonald 2017). It also may have practical value for governments and citizens in terms of fostering the resilience and sustainability of regional, rural, and remote communities and harnessing the strengths of rural communities as adaptive and innovative (Kenny et al. 2013; Simpson and McDonald 2017).

Deliberations about governance frameworks for health systems often start with discussions of centralization versus decentralization. The literature on health system reform discusses the benefits and deficits of centralized versus decentralized models of health system governance (Sreeramareddy and Sathyanarayana 2019). Adherents of centralization argue efficiencies: economic, managerial, and in terms of standardization of care. In Alberta, Canada, the Rural Health Services Review Committee (2015, 5) stated:

It is critical to recognize that cost comparisons between urban and rural regions will invariably favour urban communities. Decision making based solely on cost-per-patient criteria will result in services in rural areas being reduced or discontinued resulting in increased consolidation and centralization in urban centres.

Adherents of decentralization argue that localized governance models may enable greater consideration of factors specific to different locales in decision-making. Neo-liberal models of health system design, especially for regional, rural, and remote places, may use a hub and spoke model. However, hubs (usually a regional centre) can be far removed from the concerns of people in smaller rural and remote places (those living nearer to the spokes). This model enables the broader system, and policymakers, to effectively ignore these concerns by only employing mechanisms to enable a limited expression of the community’s voice, rather than devolution of power and control or even shared power and control down to the community-level. In other words, more devolved mechanisms for health system governance at the local level are ignored (Simpson and McDonald 2017). When these are discussed, for example, with respect to Indigenous managed health services (metro or non-metro), ongoing practices of colonialization often silence or undermine comprehensive accounts centring on Indigenous perspectives on what local governance can and should look like (Topp et al. 2022). This practice has implications, noted in part in a recent New South Wales Inquiry into regional, rural, and remote health (Legislative Council New South Wales Portfolio Committee No. 2-Health 2022), for the provision of culturally (un)safe care and (dis)empowerment of local decision-makers.

A focus of healthcare delivery in regional, rural, and remote contexts is primary care supported by secondary hospitals in some locations. The 1978 Alma Ata Declaration included participation in governance as part of the definition of primary healthcare (World Health Organization 1978). It was argued that people have both a right and a duty to participate individually and collectively in planning and implementing mechanisms that affect their health (Blacksher 2013). This participatory approach has been subsequently affirmed in a number of international agreements on primary care and the social determinants of health, including the 1986 Ottawa Charter (World Health Organization 1986). The Council of Europe (2000) has stated that “[t]he right of citizens and patients to participate in the decision making process … must be viewed as a fundamental and integral part of any democratic society.” It follows then that community participation should be enabled through the transfer of power not just voice. Further, this should not be in the context of the employment of a utilitarian model of participation but an intrinsic model. This is particularly the case for the provision of regional, rural, and remote healthcare.

Health service design has a significant impact on regional, rural, and remote communities: on access to the right health services, at the right time, in the right place, of the right type for that community or at least some members of it, on health outcomes, and on economic and social functioning (Atmore et al. 2023; McDonald and Malatzky 2023; Simpson and McDonald 2017). Many reports have identified that regional, rural, and remote communities experience health services decision-making as something that occurs from afar, in another place (the urban). Central to this is the concern that decision-makers from away, especially those who are urban-based, may not be able to consider, or even conceive of, alternative social and ethical assumptions and positions that are outside their experience and/or outside of the technocratic lens to which they are accustomed (Mullen et al. 2011).

The effects of this dynamic are compounded by the dominant portrayal of rural communities as homogenous, which, as a discursive technique, effectively controls how “rural matters” are considered (and assigned to the periphery) in the broader political milieu (Malatzky and Bourke 2016; Malatzky and Couch 2023). Regional, rural, and remote places are profoundly heterogenous (Malatzky and Couch 2023; Simpson and McDonald 2017) and the needs of these communities for different forms and focuses of health services differ (Kenny et al. 2013; Malatzky and Bourke 2016). People develop values based on their experiences as service users within a specific place context with distinct local norms (Bourke et al. 2012). These values can be coupled with an understanding of both technical aspects of healthcare and economic concerns, and of social and ethical arguments (Martin 2008) to enrich local decision-making.

Regional, rural, and remote communities often remain distanced and/or excluded from decisions that have a significant impact on their physical, psychological, economic, and socio-emotional well-being and that of their neighbours. This distancing has been reported for several decades (Kenny et al. 2013; Simpson and McDonald 2017). Rural disadvantage in respect of timely and appropriate access to health services and equity in health outcomes has been reported, also for many decades (Australian Institute of Health and Welfare 2022; Legislative Council New South Wales Portfolio Committee No. 2-Health 2022; Rural Health Services Review Committee 2015; Scheil-Adlung 2015; World Health Organization 2021). During this time-period, there have been tweaks to training, funding, and structures for regional, rural, and remote health professionals and some investments in service innovation. This has also been accompanied by the closure (designed or because of workforce sustainability issues) of some rural hospitals and some primary care services, as well as limitations on specific types of services (such as maternity services). Yet disadvantage remains. Perhaps it is time to consider reforms to governance structures in regional, rural, and remote health services using the empowerment model as a mechanism to enable justice.

## Resistance to Revisiting Governance Models

Several obstacles are associated with realizing such a vision. One, discussed in more detail above, is the dominance of neo-liberal thought with its privileging of decision-making by technocrats focused predominantly on fiscal concerns (Simpson and McDonald 2017; Wakerman and Humphreys 2013). Neo-liberalism is audit-focused (Power 2021). Lloyd et al. (2021) acknowledge the argument that participation in decision-making is framed as an ethical and democratic right, irrespective of outcomes. They then continue (2):

However, in an era of finite health service resources, robust evidence of the outcomes of public involvement is critical for health service planning. Public involvement requires investment of time and energy for the service and public participants. Financial and other costs can be significant. Participants want their contributions to make a difference, and services want good value for the resources required. Without robust evaluation of outcomes, there is a risk that public involvement is merely a tokenistic attempt to comply with government policy and accreditation standards.

While evaluation of whether mechanisms to enable participation are more than token efforts to enable the public’s voice is important, the framing of this concern is also very consistent with neo-liberal norms. Within this framing, at least in part, the legitimacy of participation is to be determined by audits of efficacy of participation (an approach based on a utilitarian model)—was there “good value” (where the who assigned to judge value is a technocrat), or indeed sufficient economic return, for the resources invested? This view can be summed up by research with health managers in New Zealand enacting this type of reform, one of whom noted: “But this is taxpayers’ money we’re all spending” (Tenbensel and Silwal 2023, 58).

Developing and implementing mechanisms to enable power, or indeed voice, can involve additional costs. Neo-liberal norms that prioritise institutionally embedded expertise may mean that any expenditure that is focused on mechanisms to enable power or voice to be provided by non-technocratic experts may be perceived as an unaffordable luxury that takes resources away from more important ends. This is particularly so given the auditability inherent in existing institutional structures. In Scotland, for example, assessments of attempts to work with communities in service design/delivery saw some service managers express reservations about the process. One noted that such processes may give the community unrealistic expectations about their power and influence in decision-making. The capacity for the exercise of power by communities was bounded by regulatory and budgetary limitations of the institutions and the accountabilities imposed on these institutions and their staff meant it was difficult for services to relinquish control or power to community (Farmer et al. 2015). In other words, participatory processes were constrained by structural factors to be more about voice rather than power. If resources and accountability are constraining factors on the capacity of managers to support or enable participation, then they may prefer to employ pre-existing participatory structures (“bolt-on” mechanisms attached to operational issues (Farmer et al. 2018, 7)), rather than mechanisms that emphasize empowerment in a strategic context. The increasing propensity of public systems to turn towards external private sector actors for this expertise, including the use of the big four consulting firms is generally based on the perception that such expertise does not exist internally and/or that private sector expertise enhances auditability; yet it also outsources potential blame. The norms of neo-liberal institutions may be designed to undermine participation as power, as institutions then lose control but retain accountability. Additionally, in a “publicly funded health system … the demands of political accountability exert a considerable force on health policy” (Tenbensel and Silwal 2023, 60).

A further tension arising from neo-liberal norms is a perception that contestability is a good thing for public policy, breeding an inherent scepticism around collaboration. This can be coupled with pragmatic concerns that effective collaboration can be dependent on the leadership of a person(s) and fails when that person(s) moves on and/or concerns about conflictual patterns of interaction in some collaborative bodies (Tenbensel and Silwal 2023). Johnston et al. (2021, 6) in an examination of health systems in rural British Columbia, Canada also found that “good relationships between providers, health authority administration, external specialist services and community members” were important for sustainable rural health. Tenbensel and Silwal (2023) found this was also required for successful network governance at the local level. They noted that neo-liberal models have fostered histories where local services have competed for scarce funding that have ingrained “transactional, arms-length and competitive relationships” (Tenbensel and Silwal 2023, 59), counter to establishing more collaborative forms of governance.

Another obstacle is the tension between the potential for local health systems governance and the purported need to ensure the maintenance of quality standards across the system (urban and regional/rural/remote) as a whole (Atmore et al. 2023). Inherently, however, standards are imbued with societal norms and are often urban-centric (Atmore et al. 2023; Simpson and McDonald 2017). Where the nature of service delivery is inherently different between rural and urban, requiring adoption of urban-generated quality standards can result in the suspension or closure of rural services because of perceived quality deficits (Atmore et al. 2023; Simpson and McDonald 2017). However, evidence of better-quality care being associated with centralization is lacking, and research from the United States indicates no significant differences in adverse events and safety between similar sized urban and rural hospitals (Atmore et al. 2023). Despite this, perceptions of a quality deficit may be used to justify a decision that is made predominantly due to fiscal concerns about the higher costs of service provision in rural areas (Bourke et al. 2012).

Adoption of urban-derived quality standards and governance norms may also have an unintended impact of stifling place-conscious innovation (Bourke et al. 2012). The privileging of metrocentric knowledges and viewpoints, built on increasing specialization and sub-specialization in healthcare, within health systems and policy means that innovations driven from rural places, based on rural knowledges and experiences, are difficult for rural actors to progress and realize (Roberts and Green 2013). Research has also indicated that for rural health systems to be successful, there needs to be a willingness by all to respond and “adapt” to “continue to deliver effective health services” (Johnston et al. 2021, 7). Given the recruitment and retention issues discussed extensively in reports and the literature more generally (Cosgrave 2020; Legislative Council New South Wales Portfolio Committee No. 2-Health 2022; Malatzky, Cosgrave, and Gillespie 2020a; Rural Health Services Review Committee 2015; World Health Organization 2021), such innovations are critical to improving access to rural health services and the health outcomes of people living outside of metropolitan places.

Concurrently, the true devolution of local governance in a neo-liberal state is restricted by the choices of governments to under-resource local levels with the expectation that individuals and communities will fill the gaps left by state and federal government disengagement (Simpson and McDonald 2017). For example, a recent report into regional, rural, and remote health service provision in the Legislative Council New South Wales Portfolio Committee No. 2-Health (2022, 2.77) raises concerns about the reliance on charity and community organizations to provide support and services associated with healthcare. In the rural context, this sense that the community are willing and able to fill the gaps may draw strength from the common stereotyping of the “rural idyll” and the construction of rural communities as self-reliant, self-sufficient, stoic, and resourceful (Malatzky and Bourke 2016; Simpson and McDonald 2017). This stereotype both shapes and justifies some of the assumptions underlying governance decisions affecting regional, rural, and remote health service design and delivery. Not every community is willing and/or has the capacity to undertake this responsibility (Barnett and Barnett 2003; Simpson and McDonald 2017). Some rural communities, as Farmer et al. (2015) and others (Tenbensel and Silwal 2023) note, will co-produce services as part of being active responsible citizens and as part of transactional relations and practices embedded in the healthcare sphere, but this can become burdensome. If unsupported or unreciprocated by the state, this can cause a legacy of mistrust (Farmer et al. 2015). As Farmer et al. (2015) have argued, the heterogenous and dynamic nature of rural communities’ contrasts with their image as necessarily highly connected and mobilized. In making this argument, Farmer et al. (2015, 65) emphasize how often those most enabled to “participate in rural civic life” belong to privileged social and economic groups and “their influence can exclude others” whose perspectives, experiences, and needs differ.

State resistance to local governance of health services by regional, rural, and remote communities may also stem from—and be justified by—stereotypes, common in highly urbanized places, that rural residents are inferior to those in metropolitan areas (Malatzky and Bourke 2016, 2018); Simpson and McDonald 2017). Here, urbanized spaces are constructed as “… progressive, where things happen and where diversity, excitement and innovation operate” (Malatzky and Bourke 2016, 161). Rural places, on the other hand, are often characterized as static or backwards looking (Bourke et al., 2012). Some of the historical roots to these stereotyping ideas can be traced to colonial thinking around metropoles as places of knowledge generation (Connell 2007). Further, rural health practice is popularly constructed as the place where health professionals who fail at or are not cut out for urban practice go (Malatzky and Bourke 2016; Simpson and McDonald 2017). The urban-centric nature of training institutions for health professionals which signals underlying assumptions about where innovation takes place serves to reinforce this perception. These dominant negative discourses centred on rurality and rural health are used to justify the metrocentric assumption that there is a lack of capacity for good governance within rural communities. This is despite examples of flourishing rural health services (Barnett and Barnett 2003), social enterprises, and businesses in some, but not all rural places, indicating motivation and capacity. Simpson and McDonald (2017) argue this stereotyping has substantial implications for justice and, as such, there is a moral imperative to deconstruct its impact on the practices of health system governance. Any such stereotyping also has the potential to undermine human rights and threaten the legitimacy of the overarching system.

## Reformulated Governance

Recently, some scholars have begun to reformulate how health systems governance should be conceptualized. For example, Siddiqi et al. (2009, 14) argues that: “governance comprises the complex mechanisms, processes and institutions through which citizens and groups articulate their interests, mediate their differences and exercise their legal rights and obligations.” Meessen (2020) notes that these types of reformulations end in broad and less normative definitions of governance and advocates a shift away from centring on government to focusing on collaboration between actors in the local space, importantly including communities. The concept of participatory democracy may be integral to this reformulation of health governance as it could broaden both the range of actors involved in governance and ensure a focus on power, as opposed to voice. This is particularly critical in regional, rural, and remote settings where access issues may be mediated by governance structures (amongst other factors).

Reforms to the health system in New Zealand attempted to move away from accountabilities resting with a single organization to accountabilities for collaborative action at the district level. This approach was described by Tenbensel and Silwal (2023) as “network governance.” As with the Scottish experience (Farmer et al. 2015), the New Zealand experience saw institutions working within existing resources (budgetary and human) to enact a transformation (Tenbensel and Silwal 2023). Committing resources is key to supporting participatory forms of governance, but more will be needed. As Tenbensel and Silwal (2023, 60) have noted: “Changing the course of local dynamics is something that will likely require much more than a specific policy initiative and is likely to require a shift in macro-institutional settings.” This goes back to power and the potential malleability of the neo-liberal structure and norms that have shaped health systems in many countries. As a qualitative study in rural British Columbia by Johnston et al. (2021, 6) articulated, successful rural health systems need autonomy—decisions that “did not require the blessing of a hierarchical, top-down system.” But this then goes to the paradox underlying place specific participatory governance reforms; that reforms to enable this broader form of governance will involve the state letting go of its reliance on power and control and valuing participatory or collaborative governance as an end in and of itself.

## Conclusion

Transitioning to this broader definition of health governance involving a true collaboration between actors could founder on the challenges outlined above. But it is also an opportunity to think more critically about governance and the role of community exercised power in determining strategic direction of local health services, especially those in rural and remote regions. This paper has argued for a re-balancing of decision-making power in health systems away from metrocentric and technocratic norms, and a fundamental re-shaping towards democratic and participatory models of governance. A health justice perspective is critical of dominant neo-liberal approaches to healthcare governance that frame the “voices” of communities as optional inputs into the system, rather than active agents in decision-making processes. In articulating this argument, the highly political nature of how we structure and govern healthcare delivery, which is imbued with value judgments influenced by neo-liberalism as well as stereotyping of rurality and rural healthcare providers and services, has been highlighted. Accordingly, we argue that moving from voice to power is particularly critical in regional, rural, and remote places where individuals and communities may feel services could and should be delivered differently to meet local needs. Given the ongoing and intractable issues with inequities in access to health services we suggestion that rethinking governance structures may be an important aspect of enabling improved access to regional, rural, and remote healthcare and achieving special justice.
